# Multiple sources and sinks of dissolved inorganic carbon across Swedish streams, refocusing the lens of stable C isotopes

**DOI:** 10.1038/s41598-017-09049-9

**Published:** 2017-08-22

**Authors:** Audrey Campeau, Marcus B. Wallin, Reiner Giesler, Stefan Löfgren, Carl-Magnus Mörth, Sherry Schiff, Jason J. Venkiteswaran, Kevin Bishop

**Affiliations:** 1 Department of Earth Sciences, Air Water and Landscape Sciences, Uppsala University, Uppsala, Sweden; 2 Department of Geography and Environmental Studies, Wilfrid Laurier University, Waterloo, Ontario Canada; 3 Climate Impacts Research Centre, Department of Ecology and Environmental Science, Umeå University, Abisko, Sweden; 4 Department of Aquatic Sciences and Assessment, Swedish University of Agricultural Sciences, Uppsala, Sweden; 5 Geology and Geochemistry, Stockholm University, Stockholm, Sweden; 6 Department of Earth and Environmental Sciences, University of Waterloo, Waterloo, Ontario Canada

## Abstract

It is well established that stream dissolved inorganic carbon (DIC) fluxes play a central role in the global C cycle, yet the sources of stream DIC remain to a large extent unresolved. Here, we explore large-scale patterns in δ^13^C-DIC from streams across Sweden to separate and further quantify the sources and sinks of stream DIC. We found that stream DIC is governed by a variety of sources and sinks including biogenic and geogenic sources, CO_2_ evasion, as well as in-stream processes. Although soil respiration was the main source of DIC across all streams, a geogenic DIC influence was identified in the northernmost region. All streams were affected by various degrees of atmospheric CO_2_ evasion, but residual variance in δ^13^C-DIC also indicated a significant influence of in-stream metabolism and anaerobic processes. Due to those multiple sources and sinks, we emphasize that simply quantifying aquatic DIC fluxes will not be sufficient to characterise their role in the global C cycle.

## Introduction

Despite rapid progress over the past decades to estimate stream DIC fluxes at the global^1, 2^, regional^3–5^ and catchment scales^6–8^, their sources still remain to a large extent unresolved. The sources of stream DIC can be diverse, ranging from biological^9–11^ to geological^2, 12^, and terrestrial^4, 13–15^ or aquatic^16–18^. While multiple studies have succeeded to define DIC sources at catchment scales^15, 19, 20^, there are fewer examples of such attempts across large landscape units^11, 21, 22^. The lack of tools to effectively characterize DIC sources across multiple catchments without requiring mass balance exercises or controlled experiments is one of the key reasons for this persistent knowledge gap. The stable carbon isotope value of DIC,^13^C-DIC/^12^C-DIC (δ^13^C-DIC) bears the imprint of multiple processes that shape the stream DIC. This makes it an attractive tool for deciphering the DIC sources. But the interpretation of large scale patterns in stream δ^13^C-DIC is known for being challenging and often results in limited outcomes. While a number of studies have analysed downstream changes in δ^13^C-DIC along large stream networks^9, 23–26^, none to our knowledge have attempted to interpret δ^13^C-DIC values across multiple individual catchments from different regions.

**Figure 1:**
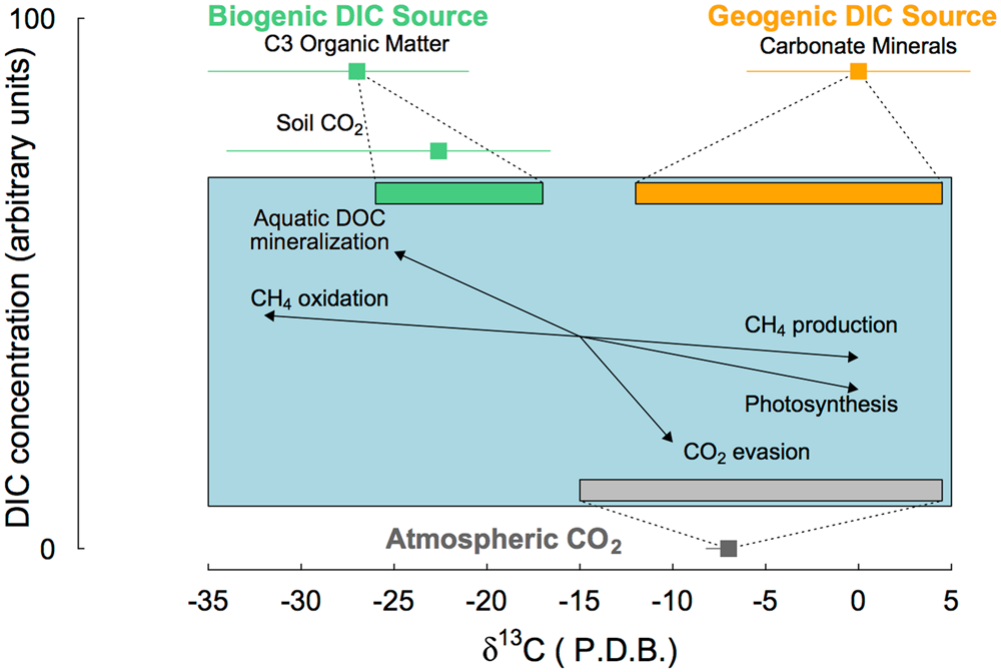
Conceptual scheme illustrating biogeochemical processes controlling stream δ^13^C-DIC values in streams, adapted from Amiotte-Suchet *et al*.^41^ and Alling *et al*.^75^. The x-axis represents the reported range of stream δ^13^C-DIC values and y-axis a gradient in DIC concentration with arbitrary units. The internationally agreed δ^13^C end-members for the biogenic (−27‰) DIC source in a C3 catchment (green square) and geogenic (0‰) DIC source (orange) as well as the atmospheric CO_2_ (−8.5‰) are represented with their documented range (coloured bars) from Coplen *et al*.^78^. The blue box represents the stream water environment, with the commonly accepted range of δ^13^C-DIC values in equilibrium with each of these three end-members (Biogenic (−26 to −18‰), Geogenic (−12‰ to 5‰), Atmospheric (−15% to 8‰), represented in coloured rectangles, along with the isotopic effect of in-stream biogeochemical processes represented as the black arrows.

The interpretation of stream δ^13^C-DIC values often begins with the very distinct isotopic values of its two major sources^27^. Biogenic DIC originates from autotrophic respiration or organic matter mineralization, with a typical δ^13^C value around −27‰ in C3 plant dominated areas^28^. When found in soil solution, this value typically increases by 1–4‰, due to dissolution and gas exchange across the soil-atmosphere interface^29–31^ (Fig. 1). In contrast, carbonate containing minerals have a typical δ^13^C value around 0‰^32^, which leads to an isotopic mixture of about −12‰, when soil respired CO_2_ is used as the acid source for the weathering reactions^33^, or even more positive values when non-carbon based acid sources are used^34^ (Fig. 1). But this simple scheme rapidly grows in complexity when accounting for the composite nature of the DIC. The δ^13^C-DIC value is the combined result of three different C components: the gaseous CO_2_ component, as well as the two ionic forms, bicarbonate (HCO^−^_3_) and carbonate (CO^2−^_3_). The evasion of CO_2_ to the atmosphere causes both kinetic fractionation as well as large isotopic equilibrium fractionation as C and its isotopes are redistributed across the different DIC components^33, 35, 36^ (Fig. 1). It is well established that streams are generally in disequilibrium with the atmospheric CO_2_^1^, with a supersaturation leading to considerable and rapid evasion of CO_2_ from stream surfaces^37^. While this process is widespread and well documented, its effect on stream δ^13^C-DIC values has only recently been described^38–40^.

Stream δ^13^C-DIC values can be shaped by more than just terrestrial export of biogenic or geogenic DIC, and atmospheric CO_2_ evasion^24, 41, 42^. Stream δ^13^C-DIC values can carry the influence of additional biogeochemical processes including: weathering of silicate minerals^26, 43, 44^, in-stream respiration^45–47^, DOC photo-oxidation^48, 49^, photosynthesis^28, 50–52^, and anaerobic metabolism^53–55^ (Fig. 1). Together, this complex mixture of sources and sinks with associated isotopic effects causes the stream δ^13^C-DIC to vary across a wide range, typically from +5‰ to −35‰ (Fig. 1). Failure to separate the different processes and influences on the δ^13^C-DIC can lead to incorrect interpretation of the sources and sinks of DIC in streams.

**Figure 2:**
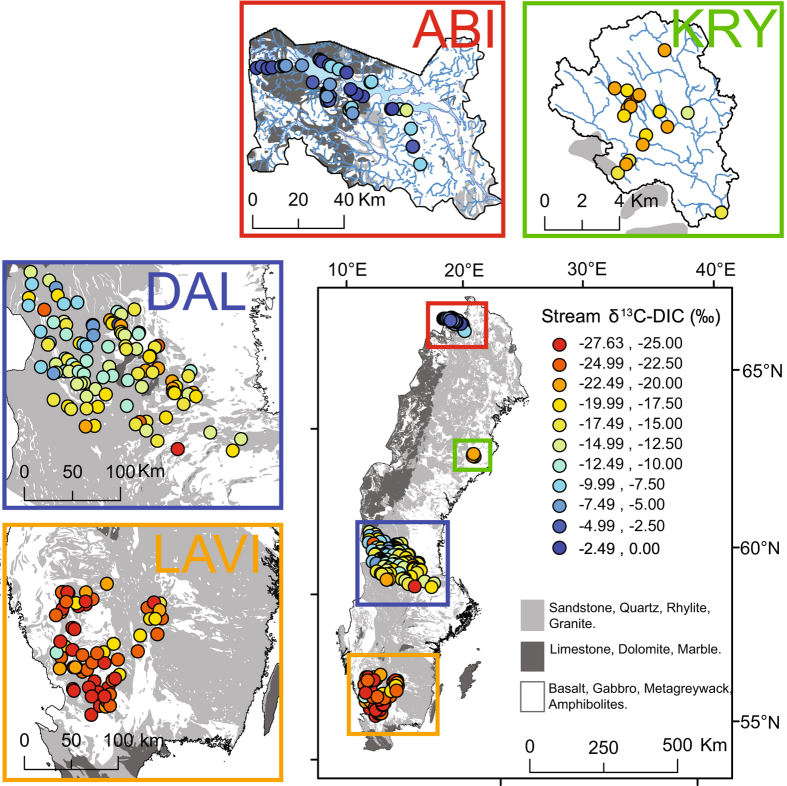
Map of sampled streams across the different regions included in this study (LAVI, DAL, KRY, ABI). Circles represent individual stream sampling locations and are colour coded according to their δ^13^C-DIC values, expressed in per mille (‰). Calcium carbonate containing bedrocks (limestone, dolomite and marble), representing a potential geogenic DIC source, are identified in dark grey, while silicate rich rocks (sandstone, quartz, rhyolite and granite) are identified in light grey and rocks that are very resistant to weathering (basalt, gabbro, metagreywacke and amphibolites) are identified in white, The map was generated using ArcMap 10.3.1 (http://www.esri.com/), with the information for the background geological map (bedrock 1:50 000–1:250 000) obtained from © Geological Survey of Sweden (SGU).

Here, we aimed to determine the sources and sinks of DIC across multiple streams and regions by exploring large-scale patterns in δ^13^C-DIC values. Stream δ^13^C-DIC data from 318 streams of Strahler stream order 1 to 5, with particular emphasis on headwater streams, were included. The streams were distributed across a large geographic and climatic range in Sweden (Fig. 2). To our knowledge, this represents the most extensive dataset on stream δ^13^C-DIC published to date. We tested a conceptual model where the stream DIC is a product of three end-members including two terrestrial DIC sources, biogenic and geogenic, as well as exchange with atmospheric CO_2_ (Fig. 1). We hypothesised that deviation from this scheme will be widespread across streams, with additional DIC sources and sinks, linked to in-stream metabolism and anaerobic processes contribute significantly to stream DIC. We explored the application of graphical mixing model techniques (Keeling and Miller-Tans plots) to identify and separate DIC sources across streams and regions (Fig. S1). We further combined these techniques with an inverse modelling approach, based on Venkiteswaran *et al*.^39^, to characterize the influence of CO_2_ evasion on the observed δ^13^C-DIC values, from which we relate the residual variance to additional DIC sources and sinks. With this approach, we were able to separate and quantify multiple processes that drive stream DIC fluxes across individual catchments and regions.

## Results

### Inter-regional patterns in stream water chemistry

The DIC concentrations ranged from 0.7 to 33.0 mg C L^−1^ across all streams, a similar range as observed for the stream DOC concentration, which varied from 0.3 to 84.4 mg C L^−1^ (Table 1). The stream DIC and CO_2_ concentration differed significantly between the regions, with the exception of KRY and LAVI. (Table 1, Table S2). The streams of KRY and LAVI both had the highest median CO_2_ concentration and lowest pH and alkalinity (Table 1). The median DIC concentrations were highest in the ABI and DAL streams, where the streams also had a circumneutral pH. The stream DOC concentration was significantly different between all four regions, with the highest median concentration observed in the streams of LAVI followed in decreasing order by the KRY, DAL and ABI regions (Table 1, Table S2). This large variability in stream C concentrations made the DOC:DIC ratio vary across four orders of magnitude (from 0.04 to 61) in the studied streams. The DOC:DIC ratio significantly decreased with altitude (m.a.s.l), following a semi-logged relationship:

1 $${\rm{l}}{\rm{o}}{\rm{g}}({\rm{D}}{\rm{O}}{\rm{C}}:{\rm{D}}{\rm{I}}{\rm{C}})=-0.004\times {\rm{A}}{\rm{l}}{\rm{t}}-2.91\quad \quad \,{{\rm{R}}}^{2}=0.61,\,{\rm{p}}\, < \,0.0001,\,{\rm{n}}=225$$

**Table 1:** Median, maximum and minimum of different stream water chemistry variables in the four different geographical regions, LAVI, DAL, KRY and ABI with *χ*², and p-value presented for Kruskal-Wallis tests (results of the Dunn’s test non-parametric pairwise multiple comparisons are presented in supplementary materials).

		LAVI n = 68	DAL n = 101	KRY n = 101	ABI n = 49
δ^13^C-DIC (‰)	Median	−24.4	−14.7	−20.5	−5.1
	Max	−10.6	−4.1	−10.8	−0.6
	Min	−27.6	−26.1	−24.9	−13.7
	Kruskal-Wallis	*χ*² = 213.15, p < 0.0001			
Calculated δ^13^C-CO_2_ (‰)	Median	−25.3	−20.1	−21.7	−15.7
	Max	−17.9	−13.0	−16.3	−9.6
	Min	−27.8	−26.2	−25.2	−20.3
	Kruskal-Wallis	*χ*² = 161.30, p < 0.0001			
δ^13^C-DOC (‰)	Median	−29.9	−28.4		−28.4
	Max	−28.0	−29.0		−29.3
	Min	−31.2	−28.1		−26.7
	n	10	10		49
DOC (mg C L^−1^)	Median	28.7	8.3	14.5	1.3
	Max	84.4	29.9	49.2	4.1
	Min	6.7	1.6	1.6	0.3
	Kruskal-Wallis	*χ*² = 221.85, p < 0.0001			
DIC (mg C L^−1^)	Median	2.6	3.8	1.7	3.8
	Max	10.3	33.0	14.9	32.0
	Min	0.7	1.1	0.6	1.2
	Kruskal-Wallis	*χ*² = 51.72, p < 0.0001			
CO_2_ (mg C L^−1^)	Median	1.7	1.22	1.34	0.48
	Max	5.3	14.9	6.63	2.76
	Min	0.3	0.46	0.59	0.30
	Kruskal-Wallis	*χ*² = 83.83, p < 0.0001			
pH	Median	5.1	6.47	5.36	7.43
	Max	6.8	7.31	7.36	8.42
	Min	4.1	4.43	3.83	7.02
	Kruskal-Wallis	*χ*² = 187.75, p < 0.0001			
Alkalinity (mmol L^−1^)	Median	0.004	0.161	0.005^*^	0.28
	Max	0.37	1.11	0.44^*^	3.00
	Min	0.00	0.00	0.00^*^	0.06
	Kruskal-Wallis	*χ*² = 153.61, p < 0.0001			
Ca^2+^ (mmol L^−1^)	Median	0.05	0.06	0.05	0.16
	Max	0.19	0.46	0.14	0.97
	Min	0.02	0.01	0.03	0.05
	Kruskal-Wallis	*χ*² = 78.05, p < 0.0001			
Ca:Na	Median	0.85	1.51	1.33	2.72
	Max	4.48	51.45	3.79	16.49
	Min	0.15	0.24	0.64	1.10
	Kruskal-Wallis	*χ*² = 91.06, p < 0.0001			

*Total alkalinity was not measured in KRY, but carbonate alkalinity was derived according to the DIC speciation and concentration.

Thus, DOC was the dominant form of dissolved C in the lowlands, while DIC was more prominent in alpine or high altitude (>450 m.a.s.l) areas (Table 1, Table S1). There was a negative relationship between stream pH and DOC concentration (mg C L^−1^) across all streams (Fig. S3).

2 $${\rm{pH}}=-0.93\times \,\mathrm{log}({\rm{DOC}})+8.06\quad \quad \quad {{\rm{R}}}^{2}=0.67,\,{\rm{p}} < 0.0001,\,{\rm{n}}=326$$

The Ca concentration was significantly different across all four regions, except in KRY and LAVI where the median Ca concentrations were also lowest (Table 1, Table S2). The median Ca concentration in the ABI streams was more than double that of the streams in LAVI, DAL and KRY (Table 1).

### δ^13^C-DIC values across streams and regions

**Figure 3:**
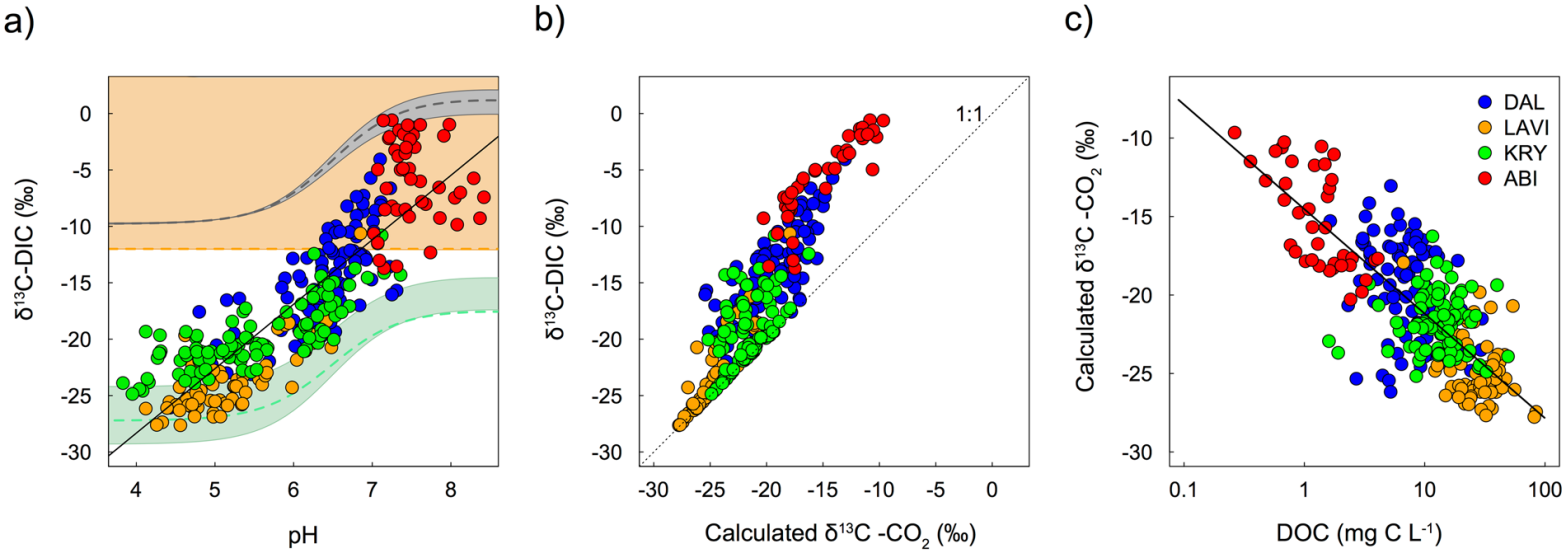
Scatterplots showing (**a**) the relationship between δ^13^C-DIC as a function of pH, with the solid black line representing the least square linear regression model, the green area representing δ^13^C-DIC in equilibrium with soil CO_2_ (−23 to −28‰), the grey area representing equilibrium with atmospheric CO_2_ (−8.5‰) the orange area representing the conventional threshold where geogenic DIC sources are considered possible, (**b**) the δ^13^C-DIC values compared with the calculated δ^13^C-CO_2_ values across all streams and regions with a dotted line representing the 1:1 ratio, and (**c**) the relationship between the calculated δ^13^C-CO_2_ values as a function of DOC concentration with the solid line representing the linear regression model. Each dot represents a different stream observation and is coloured according to its region (DAL, LAVI, KRY and ABI).

The stream δ^13^C-DIC values varied from −27.6‰ to −0.6‰, and were significantly different between all four regions, with the most negative median values found in LAVI, and the most positive values in ABI (Fig. 2, Table 1, Table S2). The stream δ^13^C-DIC values were most variable in the ABI region, with a coefficient of variation (CV) of 66%, followed by 31% in DAL, 15% in KRY and 14% in LAVI. There was a strong positive relationship between stream pH and δ^13^C-DIC across all streams (Fig. 3a):

3 $${{\rm{\delta }}}^{13}{\rm{C}} \mbox{-} \mathrm{DIC}=5.71\times {\rm{pH}}-51.17\quad \quad \quad {{\rm{R}}}^{2}=0.75,\,{\rm{p}} < 0.0001,\,{\rm{n}}=318$$

This relationship corresponded to a negative relationship between the δ^13^C-DIC values and the CO_2_:DIC ratio:

4 $${{\rm{\delta }}}^{13}{\rm{C}} \mbox{-} \mathrm{DIC}=-6.99\times {{\rm{CO}}}_{2}:\mathrm{DIC}-21.54\quad \quad \quad {{\rm{R}}}^{2}=0.68,\,{\rm{p}} < 0.0001,\,{\rm{n}}=312$$

There was a significant difference in average δ^13^C-DIC values between stream orders for the two regions including stream orders >1 (KRY p < 0.0001, and ABI, p < 0.0001 ANOVA). For both regions, the average δ^13^C-DIC values increased progressively with increasing stream order (Fig. S5). For the KRY region where stream sampling was repeated at seven different occasions, the average δ^13^C-DIC value was significantly more positive at one sampling occasion (August), but remained more similar across the other sampling occasion (Fig. S6).

### Calculated δ^13^C-CO_2_ values

The δ^13^C-DIC values could be adjusted for the influence of pH on the DIC speciation by deriving the unique δ^13^C value of one DIC component, CO_2_ in the present case (eqsS1-9, Fig. 3b). The calculated δ^13^C-CO_2_ values varied from −27.8‰ to −9.7‰, and were significantly different between all four regions (Table 1, Table S2). The calculated δ^13^C-CO_2_ followed a similar inter-regional trend as the δ^13^C-DIC values, with the most negative values found in LAVI, while the most positive values were in ABI (Table 1, Table S2). The calculated δ^13^C-CO_2_ values were most variable in the ABI region, with a CV of 21%, followed by 15% in DAL, 8% in KRY and 8% in LAVI. A significant relationship between the calculated δ^13^C-CO_2_ (‰) and pH still remained, but with a much weaker predictive power than for δ^13^C-DIC (Fig. S2).

5 $${{\rm{\delta }}}^{13}{{\rm{C}} \mbox{-} \mathrm{CO}}_{2}=-2.29\times {\rm{pH}}-34.63\quad \quad \quad {{\rm{R}}}^{2}=0.37,\,{\rm{p}} < 0.0001,\,{\rm{n}}=307$$

There was a negative semi-log relationship between the calculated δ^13^C-CO_2_ (‰) and DOC concentration (mg C L^−1^) across all streams and regions (Fig. 3c):

6 $${{\rm{\delta }}}^{13}{{\rm{C}} \mbox{-} \mathrm{CO}}_{2}=-2.89\times \,\mathrm{log}\,{\rm{DOC}}-14.52\quad \quad \quad {{\rm{R}}}^{2}=0.58,\,{\rm{p}} < 0.0001,\,{\rm{n}}=310$$

This relationship had a higher explanatory power on δ^13^C-CO_2_ than pH (R^2^ = 0.37) (Fig. S2) and spanned a gradient of three orders of magnitude in DOC concentration.

### Keeling and Miller-Tans plots

**Figure 4:**
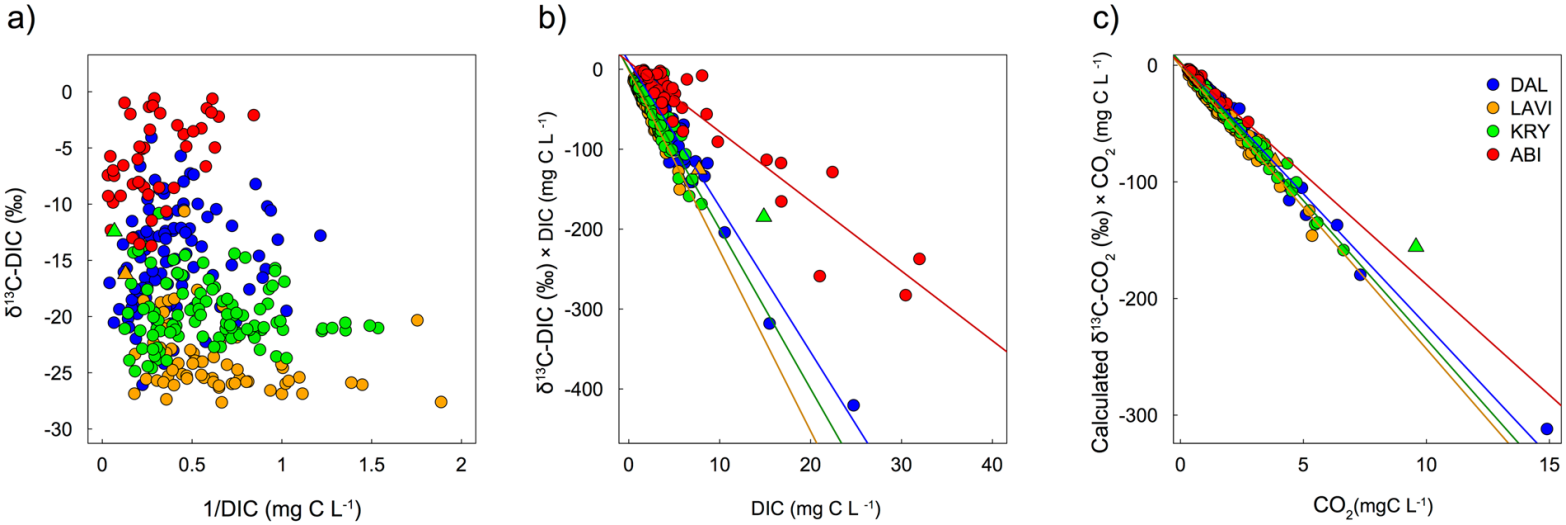
Keeling plot and Miller-Tans plot analysis for δ^13^C-DIC values (**a**,**b**) and the calculated δ^13^C-CO_2_ values (**c**). The Keeling plots (**a**) show the relationship between δ^13^C values as a function of 1/C, while the Miller-Tans plots (**b**,**c**) presents the relationship between δ^13^C-DIC × DIC as a function of DIC. The points are coloured according to their regions (LAVI, DAL, KRY, ABI). The regression lines are plotted for each individual region and follow the regional colour coding, with the equations listed in Table 2. The triangles represent stream outliers (LAVI n = 1, KRY n = 1) identified with the Cook’s distance and discussed in supplementary materials.

There was no significant relationship in the Keeling plots (δ^13^C-DIC as a function of 1/DIC), either by combining all streams or individual regions (Fig. 4a). Nonetheless, the scattering of the δ^13^C-DIC values demonstrated that streams with the highest DIC concentration (1/DIC < 1) covered the full range of δ^13^C-DIC values (Fig. 4a). In contrast, streams with the lowest DIC concentration (1/DIC > 1), generally corresponded with the most negative δ^13^C-DIC values (Fig. 4a).

There were significant relationships in the Miller-Tans plot (δ^13^C-DIC × DIC as a function of DIC concentration) for each individual region (Fig. 4b) The linear regression models were significantly different between the regions (ANCOVA, F = 57.2, p < 0.0001). The δ^13^C-DIC source value, approximated from the slope of the Miller-Tans regression models, showed two major groups of δ^13^C-DIC values, a more positive δ^13^C-DIC influence in ABI (–8.7‰), and more negative δ^13^C-DIC influences in the others, DAL (–18.2‰), KRY (–20.0‰) and LAVI (–22.6‰) (Fig. 4b and Table 2).

**Table 2:** Summary of the least-square linear regression model equations in the Miller-Tans plots for DIC and CO_2_ presented in Fig. 4b,c, with the parameters of the linear equation along with standard error in brackets for the slope, adjusted R^2^, number of observation (n) and p-values for the regression model and slope. An approximation of the δ^13^C source values can be found in the slope of the Miller-Tans equation.

Region	Equations	R^2^_adj_	n	p-value regression	p-value slope
Miller-Tans plot with δ^13^C-DIC (Fig. 4b)					
ABI	δ^13^C-DIC × DIC = 9.16–8.72 (±0.49) × DIC	0.88	43	<0.0001	<0.0001
DAL	δ^13^C-DIC × DIC = 9.70–18.18 (±0.53) × DIC	0.92	100	<0.0001	<0.0001
KRY	δ^13^C-DIC × DIC = 0.41–20.02 (±0.61) × DIC	0.92	100	<0.0001	<0.0001
LAVI	δ^13^C-DIC × DIC = −1.48–22.56 (±0.85) × DIC	0.91	67	<0.0001	<0.0001
Miller-Tans plot with the calculated δ^13^C-CO_2_ (Fig. 4c)					
ABI	δ^13^C-CO_2_ × CO_2_ = 1.83–18.97 (±0.52) × CO_2_	0.97	43	<0.0001	<0.0001
DAL	δ^13^C-CO_2_ × CO_2_ = 2.28–22.51 (±0.29) × CO_2_	0.98	100	<0.0001	<0.0001
KRY	δ^13^C-CO_2_ × CO_2_ = 2.75–23.77 (±0.27) × CO_2_	0.99	100	<0.0001	<0.0001
LAVI	δ^13^C-CO_2_ × CO_2_ = −0.66–24.25 (±0.55) × CO_2_	0.97	67	<0.0001	<0.0001
All regions	δ^13^C-CO_2_ × CO_2_ = −0.33–22.18 (±0.27) × CO_2_	0.96	251	<0.0001	<0.0001

The relationships in the Miller-Tans plot comparing δ^13^C-CO_2_ × CO_2_ as a function of CO_2_ concentration, were also highly significant for each of the individual regions (Fig. 4c). The δ^13^C-CO_2_ source values, approximated from these regression linear models, were relatively similar between regions DAL (–22.5‰), KRY (–23.8‰), LAVI (–24.2‰) and in ABI (–19.0‰), but nonetheless significantly different from each other (ANCOVA F = 24.8 p < 0.0001) (Fig. 4c and Table 2). The estimated δ^13^C-CO_2_ source for all four regions together was –22.2‰ (Table 2), which corresponded to a 6.4‰ increase relative to the median δ^13^C-DOC value (–28.7‰), determined from a subset of stream samples from LAVI, DAL and ABI (Table 1).

### Modelling of stream CO_2_ evasion

**Figure 5:**
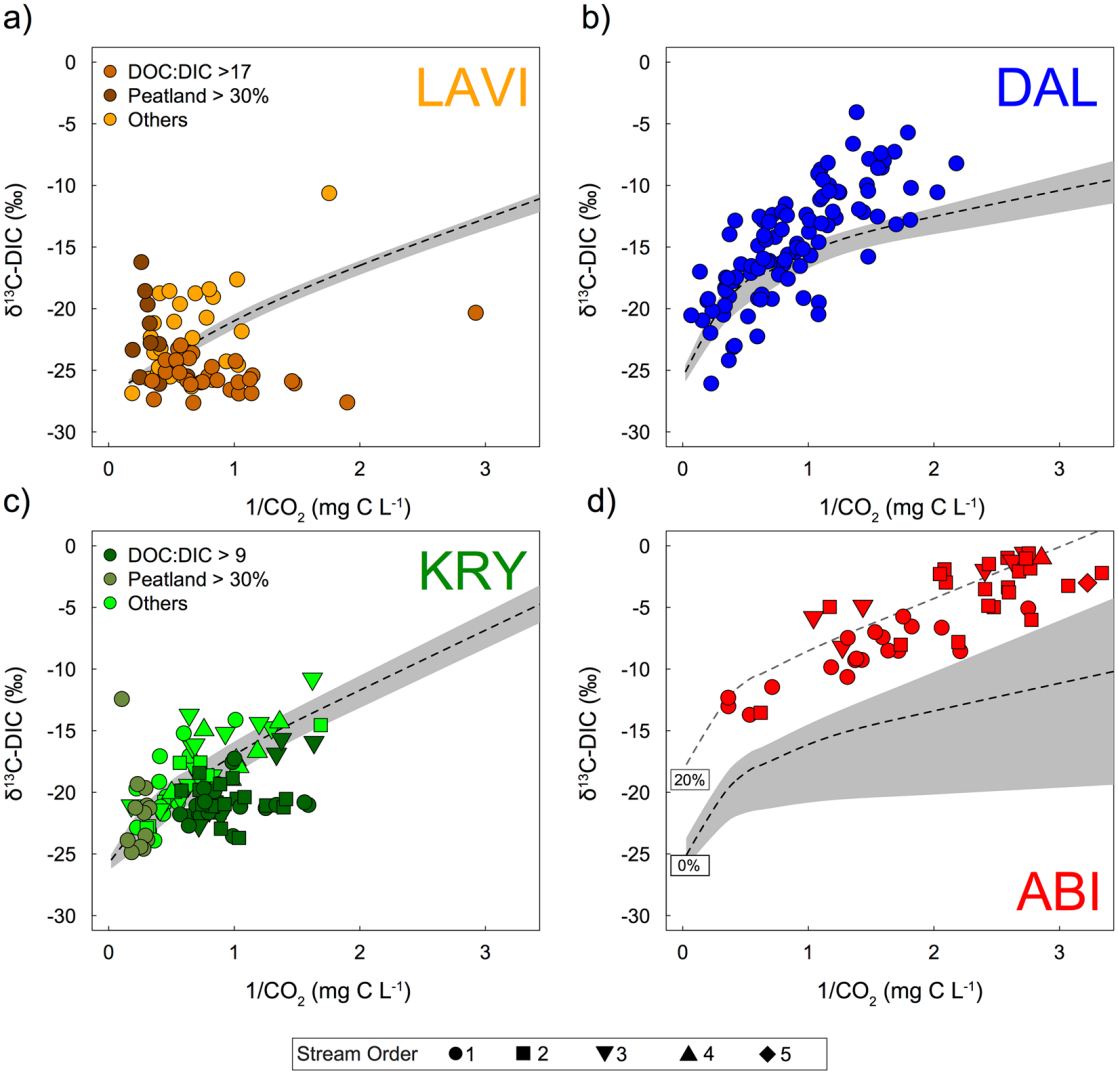
Scatterplots showing the relationship between δ^13^C-DIC and the inverted CO_2_ concentration [1/CO_2_], comparing stream observations with modelled trajectories of δ^13^C-DIC evolution with CO_2_ evasion for the streams in (**a**) LAVI, (**b**) DAL, (**c**) KRY and d) ABI^39^. The mean modelled trajectories are represented as the black dotted line with the grey area illustrating the upper and lower prediction boundaries. Each point represents a different stream observation. In the case of (**a**,**c**) certain streams were also coloured to identify streams with DOC:DIC ratios above the regional average, and peatland cover >30%. In the case of (**c**,**d**) different symbols were attributed to the Strahler stream order (1–5). In (**d**), additional curves represent the shift in modelled CO_2_ evasion with 20%, contribution of geogenic DIC source (initial δ^13^C-DIC value = −20‰).

**Figure 6:**
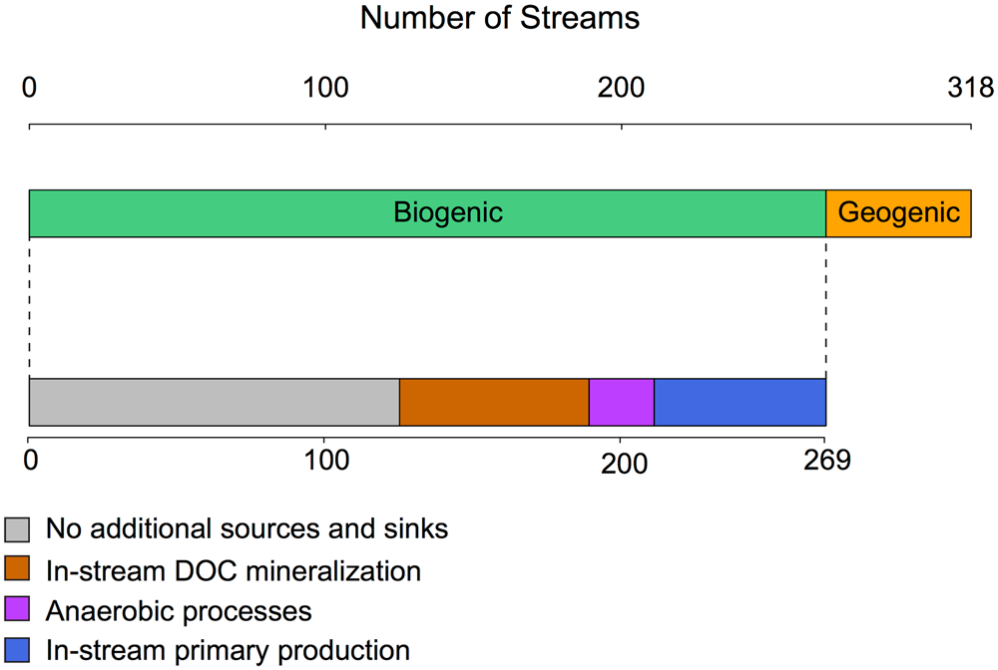
Synthesis scheme representing the identified dominant DIC sources and sinks for the studied streams (n = 318). The top bar shows the separation between streams with strictly biogenic DIC sources (n = 269) (green (DAL, LAVI and KRY)) or with a detected geogenic DIC influence (n = 49) (orange (ABI)). The bottom bar represents the streams within the category of biogenic DIC sources for which additional DIC sources and sinks are important. All streams were considered to be affected by CO_2_ evasion. The grey area represents the streams with no detectable additional influence besides CO_2_ evasion to the atmosphere (n = 126). The brown area represents streams where DOC mineralization was identified (n = 64). The purple area represents the streams likely influenced by anaerobic processes (n = 22). The blue area represents the streams possibly influenced by in-stream primary production (n = 58).

The modelled evolution of stream δ^13^C-DIC value by CO_2_ evasion of strictly biogenic DIC followed different trajectories in each region, in agreement with the observed inter-regional differences in stream DIC concentration, alkalinity and pH (Fig. 5, Table 1). Comparing the observed stream δ^13^C-DIC values with the CO_2_ evasion model showed that many stream δ^13^C-DIC values could be explained by the isotopic effect of CO_2_ evasion alone, with the largest proportion of streams found in KRY (60%) and DAL (42%), followed by LAVI (32%) and ABI (2%) (Figs 5 and 6). The proportion of stream δ^13^C-DIC observations that were more negative than the CO_2_ evasion model was highest in the LAVI region (41%), followed by a few observations in KRY (26%) and DAL (10%), but none in ABI (Figs 5 and 6). In contrast, nearly all δ^13^C-DIC values in the ABI streams (96%) were more positive (<9‰) than the evasion model. More positive stream δ^13^C-DIC values compared with the evasion model were also common in DAL (48%), LAVI (26%), and KRY (14%) (Figs 5 and 6).

## Discussion

It is well established that stream DIC fluxes play a central role in the global C cycle, but studies addressing the sources and sinks of the DIC provide divergent results. While some studies consider streams as passive conduits of terrestrially produced DIC to the atmosphere^13, 15, 56^, other have put much emphasis on the importance of in-stream biogeochemical processes modulating the DIC^9, 11, 17^. Here, we demonstrate that stream DIC sources and sinks are diverse and that both of these conceptual views are valid across different streams and regions. While in many cases, stream δ^13^C-DIC values could be explained by the combined influence of terrestrial DIC sources (biogenic/geogenic) followed by atmospheric CO_2_ evasion, there was also a large proportion of streams that deviated significantly from this model. The data suggested that in-stream DOC mineralization and primary production, as well as terrestrial or aquatic anaerobic processes, likely contributed to DIC fluxes across numerous streams and regions. Through the following steps, we were able to separate and further quantify those different processes using large-scale patterns in δ^13^C-DIC values across multiple streams.

The δ^13^C-DIC values observed across Swedish streams ranged from –27.6‰ to –0.6‰, thus covering nearly the full range of reported values for inland waters on a global scale^33, 57^. This range in δ^13^C-DIC values incorporated all three DIC end-members defined within our conceptual framework, biogenic and geogenic sources and atmospheric CO_2_ (Fig. 1). It is common practice to interpret δ^13^C-DIC values above −12‰ as indication of some degree of geogenic DIC influence^27^ (Fig. 1). Such positive values were found in 26 streams from DAL, and 45 from ABI, as well as single examples from KRY and LAVI. However, the stream geochemistry and underlying lithology of the catchments could only support the presence of geogenic sources in a number of streams from ABI and a few streams from DAL (Fig. 2, Table S1, Fig. S4)^42, 58^. This therefore highlighted some inconsistencies in the interpretation of δ^13^C-DIC values when using this simple threshold.

Theoretical trajectories showing stream δ^13^C-DIC values in equilibrium with biogenic soil CO_2_ and atmospheric CO_2_, compared with the –12‰ end member for geogenic DIC sources, demonstrated a large overlap in δ^13^C-DIC values between those three end-members, when taking into account the changes in DIC composition corresponding to pH (Fig. 3a). This demonstrates that geogenic DIC sources cannot be identified correctly by using this simple threshold for stream δ^13^C-DIC values, which assumes simple mixing of geogenic and biogenic DIC. The stream pH explained a large proportion of the variability in δ^13^C-DIC values (R^2^ = 75%). Interpreting stream δ^13^C-DIC values based solely on the direct values, and without contextualization with pH and the DIC composition, may lead to false interpretation of the processes governing stream DIC^59^. Such contextualisation can be accomplished by calculating the δ^13^C of one of the DIC components, in our case δ^13^C-CO_2_ values (eqsS1-9)^35^ (Fig. 3b), to remove the interdependence between δ^13^C-DIC values and pH. Then, other influences on δ^13^C-DIC across streams can be further disentangled, an approach that was also adopted by Mayorga *et al*.^9^ and Quay *et al*.^60^.

Using the Keeling plot analysis did not allow any clear identification of DIC sources from the stream δ^13^C-DIC values, due to the absence of significant linear relationships (Fig. 4a). This possibly occurred since equilibration between stream DIC and the atmospheric CO_2_ leads to variable background conditions depending on stream pH and alkalinity^38–40^, which violates the requirements of the Keeling plot^61, 62^ (Fig. S1). In this respect, the separation of different groups of δ^13^C-DIC values was facilitated by the Miller-Tans plot, which allows for background conditions to vary independently across observations, hence making it a more suitable technique for approximating DIC sources across multiple catchments^63^ (Fig. 4a,b). With this method, we were able to identify two groups of streams with distinct DIC sources (Fig. 4b, Table 2). The first group included the LAVI (–22.6‰), KRY (–20.0‰), and the DAL (–18.2‰) streams, with more negative slopes suggesting a prevailing biogenic influence (Fig. 4b, Table 2). While the second group, including the ABI streams (–8.7‰), revealed a clear geogenic influence, as deducted from the more positive slope (Fig. 4b, Table 2). This interpretation of the δ^13^C-DIC source values was well supported by the stream geochemistry and catchment lithology of the individual regions^42, 58^ (Table 1, Fig. 2, Table S1 and Fig. S4). The slight differences in slopes of the Miller-Tans plot for the three regions without a clear geogenic DIC influence (LAVI, KRY and DAL), likely reflected inter-regional variability in stream alkalinity, associated with their different lithologies and vegetation (Fig. 2, Table S1)^58^. The influence of geogenic DIC sources was overall low across our dataset, with biogenic sources generally dominating the DIC across Swedish streams. These results are in agreement with the latest national budget of inland water DIC export, which estimates that DIC fluxes across Swedish inland waters are predominantly driven by biogenic sources, with silicate weathering reactions rather than carbonate as the main source of alkalinity^22^.

The importance of biogenic DIC sources was further supported by the strong relationship between the calculated δ^13^C-CO_2_ values and stream DOC concentration across all streams and regions (Fig. 3c). The explanatory power of the calculated δ^13^C-CO_2_ as a function of DOC (R^2^ = 0.58) was higher than for pH (R^2^ = 0.37), indicating that this relationship could only be partly caused by organic acidity or the interdependence of pH and δ^13^C-DIC (Fig. S2). A similar relationship was reported from large river systems and associated to similar drivers^25^. These results further support that changes in soil and inland water DOC concentration, reported across many areas of the northern hemisphere, may affect not only the magnitude of aquatic CO_2_ emissions^17^ but also the source of stream DIC.

The Miller-Tans analysis of the calculated δ^13^C-CO_2_ values demonstrated that the source of DIC, when adjusted for differences in stream pH, was highly similar between regions (inter-regional differences being <3‰; Fig. 4c). The approximated source of δ^13^C-CO_2_ across all four regions (–22‰; Table 2), represents a significant deviation from the isotopic composition of stream water DOC, although subject to a certain degree of uncertainty (median δ^13^C-DOC = −28.7‰; Table 1). This indicated that in-stream DOC mineralization was unlikely to be the main source of stream DIC across all streams. This value is instead more representative of soil pore water CO_2_, which is typically more positive than the directly respired CO_2_ (1–4.4‰) due to gas exchange across the soil-atmosphere interface^30, 31, 64^ (Fig. 1). Similar δ^13^C-DIC values were also observed from a subset of soil water samples in KRY (δ^13^C-DIC −22.7 ± 1.1‰, pH 5.0 ± 0.4, n = 38) and in a catchment just north of the LAVI region (δ^13^C-DIC −23.5 ± 2.2‰, pH 5.5 ± 0.5, n = 65) (Campeau *et al*., submitted July 2017.), further supporting our interpretations of the approximated CO_2_ source from the Miller-Tans plot (Fig. 4c). These results suggest that soil respiration, rather than direct in-stream DOC respiration, was the dominant source of stream DIC across all four regions, in spite of major differences in landscape geomorphology, productivity and climate, as well as the presence of carbonate containing minerals mainly in the ABI region (Table S1).

All streams were supersaturated in CO_2_ relative to the atmosphere, showing that CO_2_ evasion occurred in all streams. Through an inverse modelling technique, we were able to describe the isotopic effect of CO_2_ evasion on the δ^13^C-DIC values within each region, assuming strictly biogenic soil DIC sources and considering interregional differences in pH and alkalinity^39^ (Fig. 5). We used the results of the CO_2_ evasion model to quantify residual variation in the stream δ^13^C-DIC values, which enabled the identification and quantification of additional stream DIC sources and sinks. We found that although CO_2_ evasion occurred across all stream observations, its isotopic effect could only fully explain the observed δ^13^C-DIC values in about half of the streams (n = 126) (Figs 5 and 6). This suggested that additional DIC sources and sinks likely influenced the δ^13^C-DIC values in a significant number of streams.

Nearly all streams in the ABI region had more positive δ^13^C-DIC values than predicted by the CO_2_ evasion model, given the model’s assumption that DIC sources are strictly biogenic. This supported well our interpretation of the geogenic DIC influence in a number of streams in this region. We determined that CO_2_ evasion, if coupled with variable contribution of geogenic DIC input (~20%), could explain most of the observed stream δ^13^C-DIC values in the ABI region. More positive δ^13^C-DIC values compared with the CO_2_ evasion model also occurred in streams located in LAVI, KRY and DAL (n = 58), regions, where geogenic DIC influence was not identified. We interpreted the δ^13^C-DIC values in these streams to be possibly affected by in-stream primary production, especially in conditions with low stream CO_2_ concentrations (<2 mg C L^−1^) (Figs 5 and 6). Photosynthesis progressively increases the stream δ^13^C-DIC value by taking up stream DIC^50–52^. We estimated that the observed deviation in stream δ^13^C-DIC from the CO_2_ evasion model, ranging from 1 to 9‰, could reflect the consumption of 4–31% of the DIC pool by in-stream primary production. While it cannot be excluded that photosynthesis also influenced some of the stream’s δ^13^C-DIC values in the ABI region, this influence could not be separated from the geogenic DIC contribution.

Streams with more positive δ^13^C-DIC values along with elevated CO_2_ concentrations (>2 mg C L^−1^) (n = 33) generally corresponded to catchments with the largest proportion of peatlands (Figs 5 and 6). Peatlands export large quantities of CO_2_ and CH_4_ to streams^5, 6, 65^ and are fuelled by anaerobic processes that are known to dramatically alter the δ^13^C-DIC values^53–55^ (Fig. 1). We estimated that the observed deviation in δ^13^C-DIC values from the CO_2_ evasion model for this category of streams, which ranged from 1–11‰, could have resulted from a 2–31% production of DIC through acetoclastic methanogenesis, or alternatively a 1–22% consumption of DIC through hydrogenotrophic methanogenesis. These anaerobic pathways cannot be clearly separated based solely on the stream δ^13^C-DIC values, thus our estimates are subject to a large uncertainty. Interestingly, two δ^13^C-DIC observations falling within this category of streams were identified as outliers from the regional δ^13^C sources determined from the Miller-Tans plot for LAVI and KRY (Fig. 4a,b). This indicated that although DIC in those streams was supported by biogenic sources, the increase in δ^13^C-DIC values generated by anaerobic processes could easily be interpreted as geogenic influences. Since peatlands cover about 15% of the Swedish landscape, as well as a large proportion of northern latitudes^66, 67^, this influence on stream δ^13^C-DIC may be widespread.

Several δ^13^C-DIC observations were more negative than predicted by the CO_2_ evasion model (n = 92) (Figs 5 and 6). These streams typically had a higher DOC:DIC ratio than the average stream (Fig. 5). In-stream DOC mineralization can supplement stream CO_2_ with an isotopic composition close to that of the DOC^47, 68^, in our case −28.7‰ (Table 1), which is more negative than the soil water CO_2_. In-stream DOC mineralization can thus potentially mask the isotopic effect of CO_2_ evasion by maintaining more negative δ^13^C-DIC values despite gas loss. The DIC produced by DOC mineralization can be an even larger source of ^12^C if produced via photochemical processes or using autochthonous OC fractions, since these processes can target molecules that are ^12^C-enriched within the bulk DOC pool^48, 49, 69^.We estimated that direct in-stream DOC mineralization could contribute to 7 to 90% of the DIC in this category of streams, with an overall average of 39%, based on the residual variation in δ^13^C-DIC (ranging from 1 to 10‰; Figs 5 and 6). For these particular streams, we estimated that such contribution to the DIC pool would represent the mineralization of 1 to 50% of the available stream DOC pool.

The contribution of in-stream processes to DIC fluxes has been demonstrated to increase along fluvial networks^11^, potentially as a consequence of changes in water residence time^70^. The downstream increase in δ^13^C-DIC values in the KRY and ABI regions, where stream orders up to 5 were included, also suggested changes in processes controlling DIC along fluvial networks (Fig. 5, Fig. S5). In low order streams, terrestrial DIC sources are often considered to exceed the aquatic sources^13, 15, 56^. Although this was the case in many of our studied streams, it was evident that DIC in a number of streams was also fuelled by aquatic processes. Seasonality is also recognised as an important modulator of in-stream processes, an aspect that cannot be clearly addressed within our dataset. Nonetheless, the potential influence of seasonality was suggested by the significant increase in stream δ^13^C-DIC values during the summer within the KRY and ABI regions, only regions where measurements were repeated across different periods (Fig. S6)^42^.

Taken together, our results demonstrate that stream DIC across Sweden arises from multiple sources and sinks. Simply accounting for terrestrial DIC fluxes and atmospheric CO_2_ evasion will not fully capture the complex role of streams in the global C cycle. Through an analysis of δ^13^C-DIC values across multiple streams and regions, we established that soil respiration was the predominant DIC source across all regions, rather than aquatic processes. The influence of geogenic DIC sources was overall low across these regions. While some of the stream δ^13^C-DIC values could be fully explained by CO_2_ evasion to the atmosphere, other streams appeared to be influenced by secondary sources and sinks, linked to in-stream metabolism and anaerobic processes either in streams or connecting soils. These additional processes made a significant contribution to the stream DIC as well as its isotopic composition. Future studies should aim to combine budgets of stream DIC fluxes with determinations of sources and sinks. Such attempts can be supported by the interpretation of large-scale patterns in δ^13^C-DIC values. The rich information contained in δ^13^C-DIC can benefit our understanding of terrestrial and aquatic C transformation processes, but it can also be misleading if interpreted too simply. The systematic approach demonstrated here made it possible to identify dominant DIC sources across different regions, as well as to quantify additional DIC sources and sinks for individual streams. This method has proven valuable in the boreal context, but its applicability should be tested in other more complex and diversified settings.

## Methods

### Sampling Design

The study is based on a total of 326 water chemistry measurements from 236 individual streams distributed among four contrasting geographical regions (LAVI, DAL, KRY and ABI) following a 1500 km long latitudinal gradient across Sweden (Fig. 2). The sampling design in the LAVI and DAL regions consisted of synoptic surveys of headwater streams (Strahler stream order 1). There were a total of 68 and 101 sampled streams in each region respectively (Fig. 2 and Table S1). The LAVI region is located in the south-west coast of Sweden and covers four different river catchments, Lagan, Ätran, Viskan, and Nissan, together covering an area of (14 700 km^2^) (Table S1). The DAL region is located in central Sweden in the Dalälven river catchment and covers a total area of 29 000 km^2^. The catchment area of each stream’s sampling point in LAVI and DAL averaged 1.8 km^2^ (from 0.2 to 6.2 km^2^). The streams in LAVI and DAL were visited on one occasion, in June 2013 and 2014 respectively, during periods of hydrological base flow conditions. Stream sampling was conducted within two weeks for both regions with the aim of reducing influences from variability in stream flow and climatic conditions. The streams were selected using a random statistical selection of headwater streams based on the following three main criteria; 1) streams were of stream order 1, but with a total stream length exceeding 2500 m in order to avoid ephemeral streams; 2) selected catchments did not contain lakes, urban areas or more than 5% agricultural land; 3) the streams were located within 0.5 km of accessible roads. This selection process provided a statistically representative sampling of a variety of land cover types in each region (e.g. abundance of wetlands, tree species, catchment morphology and geology). Further information about the random statistical selection of the headwaters streams can be found in Wallin *et al*.^58, 71^, and Löfgren *et al*.^58, 71^.

Stream sampling in the KRY region was conducted as part of a regular sampling program within the Krycklan Catchment Study (KCS), a boreal catchment that has been intensively studied since the 1980s with a focus on hydrology, biogeochemistry and stream ecology^72^ (Fig. 2). The stream water sampling in KRY was distributed among 18 different streams that were visited on three occasions in 2006 (June, August and November) and on four occasions in 2007 between late April and late May, for a total of 108 individual samples (Table S1). The streams in KRY comprise a range of stream sizes, from stream order 1 to 4 with catchment areas ranging from 0.04 to 67.9 km^2^ and with various land cover compositions (i.e. forest, mires, lakes) (Table S1). Part of the data from the KRY sampling has been published in Venkiteswaran *et al*.^39^.

Stream sampling in the sub-arctic ABI region included 49 different streams scattered around Lake Torneträsk and were visited on one occasion in mid-September 2008 (Table S1). The data from the sampling in ABI has been published in Giesler *et al*.^42^. The streams in ABI included a wide range of stream sizes, from stream order 1 to 4 and with catchment areas of 0.34 to 565 km^2^ (Table S1). Roughly one third of the ABI streams are located above the tree line, while the remaining two-thirds are found in catchments with a mixture of tundra and sub-alpine birch forest.

### Stream water chemistry analysis

Stream water samples were collected for analysis of basic chemistry (pH, alkalinity, major cations and anions) and dissolved carbon concentrations (DIC, DOC, and CO_2_). The stream water samples were collected approximately 10 cm below the stream surface and as far away from the stream banks as possible. Stream water DIC and CO_2_ concentration was measured using the acidified headspace method in LAVI, DAL and KRY^65, 73^. More details about the sampling method can be found in Wallin *et al*.^58^ for the KRY samples, and in Wallin *et al*.^71^ for the LAVI and DAL samples. In the ABI streams, the CO_2_ concentration was determined with the headspace equilibration technique, which is detailed in Giesler *et al*.^42^.

Stream water samples for DOC concentration analysis were collected in acid-washed high-density polyethylene bottles and stored refrigerated until analysis. All samples were acidified and sparged to remove inorganic carbon prior to analysis. The samples were analysed within two weeks after collection using a Shimadzu TOC-V + TNM1, except for the samples in ABI which were analysed with a Shimadzu TOC-VcPH total organic carbon analyser. Previous analysis has shown that the particulate fraction of TOC on average is less than 0.6% in boreal streams, indicating that DOC and TOC are essentially the same.

Samples for analysis of pH in DAL, LAVI and KRY were collected in 50ml high-density polyethylene bottles, which were slowly filled and closed under water in order to avoid pockets of gas in the bottle. The pH samples were analysed using a using an Orion 9272 pH meter equipped with a Ross 8102 low‐conductivity combination electrode with gentle stirring at ambient temperature (20 °C) of the non-air equilibrated sample with an accuracy of ±0.1 units. For the ABI samples, both alkalinity and pH were measured using a Metrohm Aquatrode Plus (6.0257/000) pH electrode (Metrohm AG, Switzerland). Alkalinity was calculated from back tritration, i.e the difference in sample volume and amount of NaOH and HCl used to titrate to pH 4.0 and back-titrate to pH 5.6. Stream water calcium (Ca^2+^), magnesium (Mg^2+^), sodium (Na^+^) concentration in all streams were determined by inductively coupled plasma atomic emission spectroscopy (ICP-AES) (Varian Vista Ax Pro). Cation concentrations are expressed without their respective charges throughout the text for simplicity. The analytical methods for determining alkalinity and base cations in the LAVI and DAL samples are accredited by the Swedish Board for Accreditation and Conformity Assessment (www.swedac.se) and follow the Swedish standard methods. All base cation concentrations were adjusted for atmospheric deposition following^74^.

### Stable isotope composition analysis

In LAVI and DAL, the samples for δ^13^C-DIC analysis were collected with a 100 ml glass vial, filled completely with stream water and closed airtight with a rubber septum below the water surface. One ml of highly concentrated ZnCl_2_ solution was injected in each sample directly after sample collection, in order to stop any further biological process. In ABI, the samples were initially collected in a 1-L bottle in the field, from which a 4mL subsample was collected in the lab and transferred into 12mL pre-flushed N_2_ septum-sealed glass vials (Labco Limited). The sampling procedure was similar for the KRY stream, except that the stream water sample was directly injected into the pre-treated 12ml glass vial. All samples were stored cold and dark until analysis. Prior to analysis, each δ^13^C-DIC sample was injected with phosphoric acid in order to convert all DIC species to CO_2_(g). Stream water δ^13^C-DOC was measured for a subset of randomly selected streams in LAVI, DAL and ABI regions, using a 500ml dark bottle filled with stream water and filtered at 0.7 μm once in the lab. Prior to analysis, the DOC samples were converted to graphite by Fe/Zn reduction and combusted to CO_2_. In DAL, the samples for δ^13^C composition were analysed using Gasbench II and a Thermo Fisher Delta V mass spectrometer. The LAVI samples were analysed by standard off-line dual-inlet IRMS techniques. The analytical instrumentation for the stream samples from ABI and KRY (only those sampled in 2007) consisted of a Gasbench II and a Thermo Finnigan MAT 252 mass spectrometer. The 2006 samples from KRY were analysed on a Europa Scientific Ltd, ANCA TG system, 20–20 analyser. The δ^13^C values are given in terms of deviation from the standard *Vienna Pee- Dee Belemnite* (VPDB) in per mille. The repeated measurements of the standard indicated a standard deviation below 0.2‰ in each regional sampling. Further information on the ABI and KRY regional stream sampling can be found in Geisler *et al*.^42^ and Venkiteswaran *et al*.^39^, respectively.

### CO_2_ evasion model and quantification of additional DIC sources and sinks

The influence of CO_2_ evasion on the streams’ δ^13^C-DIC values was modelled for each individual region following Venkiteswaran *et al*.^39^. The time-forward model is built around several assumptions 1) no carbonate dissolution contributes to the DIC pool, 2) in-stream processes are negligible, 3) carbonate alkalinity is conserved in the system, and 4) the contribution of organic acids to total alkalinity does not affect carbonate alkalinity as CO_2_ is evaded. The model was run iteratively over 90 different runs to solve for the combinations of initial DIC concentration and pH that best fit the range of observed stream DIC concentration, pH, and δ^13^C-DIC values. Initial conditions for the region-specific modelled fits are listed in supplementary material (Table S3). The initial δ^13^C-DIC values were assumed constant across all iterations at −26‰, thus representing a DIC originating from direct mineralization of organic matter or soil respiration (−27‰). Inflow of additional soil-respired CO_2_ along the stream reach would, in theory, shift the δ^13^C-DIC values back towards the origin of the CO_2_ evasion model trajectory, assuming that soil DIC characteristics are similar to across the entire catchment. We consider this to be fairly reasonable assumption based on the small size of these low order stream’s catchment. More information about the modelling approach can be found in Venkiteswaran *et al*.^39^.

The residual variation between the observed stream δ^13^C-DIC values and the upper or lower boundaries of modelled δ^13^C-DIC values from the CO_2_ evasion model were analysed in order to quantify additional DIC sources and sinks. For the streams where δ^13^C-DIC values could be explained by the CO_2_ evasion model (residual variance <1‰), we assumed that no additional DIC sources and sinks affected stream DIC other than CO_2_ evasion (Figs 5 and 6). This residual variation in δ^13^C-DIC was separated in different categories that matched the direction of the different isotopic effects taken into consideration (Fig. 1). The contribution of in-stream DOC mineralization was calculated using a two-component mixing model, assuming mixing with −28.7‰, which represents the median δ^13^C-DOC observed across a subset of streams from this dataset and potentially incorporates both autochthonous and allochthonous OC. The contribution from carbonate weathering sources (i.e. geogenic C) was estimated with a similar approach, but assuming dissolution of carbonate rocks with a value of 0‰, forming an even mixture with soil-respired CO_2_ (δ^13^C-DIC −12‰). This respiratory contribution would be lower if non-carbon based acid were utilized^34^. Several biological processes influencing the DIC pool and its isotopic composition can be described through a Rayleigh approach:

7 $${{\rm{\delta }}}^{13}{{\rm{C}}}_{{\rm{obs}}}={{\rm{\delta }}}^{13}{{\rm{C}}}_{{\rm{source}}}+{10}^{3}({\rm{\alpha }}-1)\,\mathrm{ln}({\rm{f}})$$

where δ^13^C_obs_ is the observed stream water δ^13^C-DIC value, δ^13^C_source_ is the δ^13^C values is the substrate, or that predicted by the CO_2_ evasion model in our case, α is the isotopic fractionation factor and f is the C flux required to explain the observed δ^13^C-DIC values.

In the case of DIC uptake by primary production, we applied an α_pp_ = 0.975, following Alling *et al*.^75^. In the case of, DIC consumption by hydrogenotrophic methanogenesis we applied a range of isotope fractionation factors α_hm_ = 1.055 to 1.085, following^76^, and used the δ^13^C-DIC_source_ value approximated for each region from the Miller-Tans analysis. Alternatively, DIC production through acetoclastic methanogensis was estimated with α_am_ = 1.040 to 1.055^76^ and δ^13^C_source_ representing the average δ^13^C-DOC value reported from this dataset.

The quantifications described above assume that deviations in δ^13^C-DIC from the CO_2_ evasion model are driven by a single dominant process, but it is likely that δ^13^C-DIC is influenced simultaneously by multiple processes. Since those processes cannot be clearly separated here, we acknowledge that our estimates are likely conservative.

In addition, the potential influence of CH_4_ oxidation on δ^13^C-DIC values was not considered here. Its isotopic effect could maintain more negative δ^13^C-DIC values despite CO_2_ loss by evasion and mimic in-stream DOC mineralization (Fig. 1). Although CH_4_ oxidation is a source of highly negative δ^13^C-DIC^53, 76, 77^, we estimated that the mass of CH_4_ in streams or soil waters was likely insufficient to fully explain the patterns in δ^13^C-DIC across these streams.

### Statistical Analysis

The non-parametric Kruskal-Wallis test was used to determine inter-regional statistical differences in water chemistry. The Dunn’s test was used for pairwise multiple comparisons of variables across the different regions. Coefficients of variation were presented to express a uniform spread relative to the mean values. Correlation coefficients were given for the Kendall correlations. Ordinary least square linear regression models were performed, for example in the Keeling and Miller-Tans plots. In alternative cases, geometric mean functional relationships were performed in situations where the relationship between the two variables was considered to be symmetrical, for example between pH and DOC concentration. Those relationships were considered significant when p-values were <0.05. Outliers in those regression models were identified using the Cooks distance, which allows for weighting the influence of each observation on the regression model. Stream observations were considered as outliers when the Cooks distance was >4, which occurred for single observations in the LAVI (*D* = 4.9) and KRY (*D* = 16.2) regions respectively. Those sites were removed from the approximation of the δ^13^C source value using the Miller-Tans plot and were identified with triangles (Fig. 4). Analyses were performed using R Core Team (2013). R: A language and environment for statistical computing. R Foundation for Statistical Computing, Vienna, Austria. URL http://www.R-project.org/.

### Data Availability

The datasets analysed during the current study are available from the corresponding author on reasonable request.

## Electronic supplementary material

Supplementary Information
