# Supplementary material for: Multiple sources and sinks of dissolved inorganic carbon across Swedish streams, refocusing the lens of stable C isotopes
Source: Sci Rep. 2017 Aug 22;7:9158. doi: 10.1038/s41598-017-09049-9 (PMC5567220; doi:10.1038/s41598-017-09049-9)
Supplement: Supplementary file 1 — Supplementary Information [file 41598_2017_9049_MOESM1_ESM.doc]

**Supplementary Information**

**Multiple sources and sinks of dissolved inorganic carbon across Swedish streams, refocusing the lens of stable C isotopes.**

Audrey Campeau [[1]](#footnote-2)*, Marcus B. Wallin ^1^, Reiner Giesler ^3^, Stefan Löfgren ^4^, Carl-Magnus Mörth ^5^, Sherry Schiff ^6^, Jason J. Venkiteswaran ^2^, Kevin Bishop ^1,4^.

## Section 1: Calculating δ^13^C values within the carbonate equilibrium system

The δ^13^C-DIC value represents the sum of δ^13^C values of each of its three components, CO_2_, HCO_3_ and CO_3_ :

δ^13^C -DIC × [DIC]= δ^13^C-CO_2_ × [CO_2_]+ δ^13^C-HCO_3_ × [HCO_3_]+ δ^13^C-CO_3_ × [CO_3_] *(eq.S1)*

The δ^13^C value of each carbonate species within the DIC pool can be derived from the δ^13^C-DIC value based on temperature dependent equilibrium fractionations ^1-3^. This can help to further develop the analysis without discussing the effect of carbonate-equilibrium fractionation on the patterns in δ^13^C-DIC values. The enrichment factors across the carbonate species are described by Zhang et al., 1995 ^1^ as follows:

ε bg = ε _HCO3(aq)_-_CO2(g)_ = – 0.1141 × *Tc* + 10.78 (‰) (eq S2)

ε dg = ε _CO2(aq)-CO2(g)_ = + 0.0049 × *Tc* – 1.31 (‰) (eq S3)

ε db = ε _CO2(aq)-HCO3_= ε dg – ε bg (eq S4)

ε cg = ε _CO3(aq)_-_CO2(g)_ = – 0.052 × *Tc* + 7.22 (‰) (eq S5)

ε cb = ε _CO3(aq)_-_HCO3(aq)_ = ε cg - ε bg / 1+ ε db × 10^-3^ (‰) (eq S6)

It is worth noting that those enrichment factors are given relative to CO_2_ in the gas phase and other studies have identified variable degrees of fractionation between CO_2(aq)_-CO_2(g)_ ^4^. The δ^13^C values of each carbonate species are calculated using the isotopic fractionation across carbonate species at stream temperature (eq S2-6) and pH, which sets the distribution of the DIC species.

(eq S7)

(eq S8)

(eq S9)

It is important to specify that the calculated δ^13^C-CO_2_ values can diverge significantly from the field δ^13^C-CO_2_, although the current literature does not allow to clearly understand the dynamics between the two (Billett & Garnett, 2010). These calculations can be used to filter the influence of pH and DIC speciation on the δ^13^C-DIC values, an aspect that is clearly important when incorporating stream with various chemical properties. This approach was also employed by (Mayor*ga et a*l., 2005, Qu*ay et a*l., 1992) to deal with similar issues, and also emphasized in (Mook & Tan, 1991).

## Section 2: Graphical tools and modelling for interpretation of δ^13^C source and fate

The Miller-Tans ^5^ and Keeling plots ^6^ were used to explore δ^13^C-DIC values and identify DIC source and fate in streams. Both graphical techniques are based on the principle of conservation of mass and assume mixing of isotopically distinct C sources (eq11)

δ^13^C_obs_ × C_obs_ = δ^13^C_S_ × C_S_ + δ^13^C_B_ × C_B_ (eq S10)

Where (δ^13^C_obs_) and (C_obs_) are the observed C isotopic composition and concentration respectively in each sample, resulting from a mixture of the C isotopic composition and concentration of the source “S”, for example biogenic or geogenic DIC source (δ^13^C_S_ × C_S_) and background “B” (δ^13^C_B_ × C_B_), represented in the atmospheric CO_2_.

This principle, if re-written as a linear relationship (y=a×x+b) is expressed as equation S11 in terms of the Keeling plot regression, and equation S12 in the Miller-Tans plot regression.

δ^13^C_obs =_ C_B_ (δ^13^C_B_ –C_S_) × (1/ C_obs_) + δ^13^C_S_ *(eq S11)*

δ^13^C_obs_× C_obs =_ δ^13^C_S_ × C_obs_ - δ^13^C_B_ (δ^13^C_B_ – δ^13^C_S_) *(eq S12)*

The δ^13^C_s_ is found in the intercept of the linear regression in equation S11 or in the slope of the linear regression in equation S12. In practice, the differences between the two regression models imply that the stream δ^13^C-DIC value in equilibrium with atmospheric CO_2_ (δ^13^C_B_ × C_B_), must remain fixed across observations in equation S11. However, this requirement can be disregarded in equation S12 since the δ^13^C_B_ × C_B_ is found in the residuals variation of the regression line (Figure S1 presents a graphical illustration of the two methods). As such, the Miller-Tans plot technique is particularly suitable for approximating the δ^13^Cs when including observations from multiple catchments that have different pH and alkalinity and have undergone various degrees of CO_2_ evasion. Both models assume linearity, with simple mixing of the two C sources/sinks without further fractionation processes ^7-9^. These assumptions can be violated in stream waters since kinetic fractionation processes occur when CO_2_ is outgassed from the stream water and other in-stream biogeochemical processes may be involved as well. Consequently, the interpretation of the δ ^13^C_S_ derived from these mixing equations must be made with care.

**Figure S1:** illustrations of the Keeling (left) and Miller Tans (right) graphical techniques, adapted from Pataki et al, 2003 ^7^. The Biogenic and Geogenic DIC sources are represented as the green and orange sections in each figure, along with the Atmospheric CO_2_ shown as the grey sections. Each figure illustrates a hypothetical set of δ^13^C-DIC values and DIC concentration observations in stream water (δ^13^C_obs_, C_obs_), represented as white circles. The hypothetical least square linear regression models, given in the text as equation S11 for the Keeling and equation S12 for the Miller-Tans, are represented graphically by the dotted lines. The hypothetical δ^13^Cs (-22.5‰) is represented in the intercept of the Keeling plot regression, but in the slope of the Miller-Tans regression. The hypothetical background is represented as the black circle falling in the Atmospheric CO_2_ section in the Keeling plot, but as the residuals of each observation in the Miller-Tans plot.

**
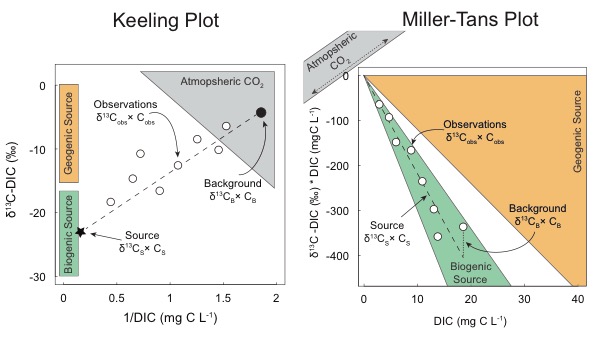
**

**Section3: Regional Landscape and Climatic Characteristics**

The LAVI region is located in the southwest part of Sweden and comprises four major rivers, the Lagan, Ätran, Viskan and Nissan, together covering a total catchment area of nearly 15 000 km^2^ (Figure 2). The region has a relatively flat topography (average elevation 167 (±71) m.a.s.l), is dominated by temperate-mixed forest (61%) and has a bedrock composed predominantly of granitic rocks (87%) (Table S1).

The DAL region is located in the central part of Sweden and is delimited by the River Dalälven catchment, which covers nearly 30 000 km^2^. The DAL region covers a wide gradient in topography, ranging from 1321 m.a.s.l. in the western part of the region, down to sea-level at the east coast of Sweden. The bedrock in the western part of the region is predominantly composed of silicate-rich sandstones and rhyolites, while the eastern part mostly consists of granite. The soils southeast of the limestone ring around Lake Siljan contains some limestone relicts transported during the last glaciation (Figure 2). The DAL region is dominated by boreal forest (67%) with a high portion of peatlands, averaging 9% of the landscape (Table S1).

The KRY region contains a range of ecosystems (forest, mires and lakes) and geomorphology, despite being the smallest region covered by this study (68 km^2^). For example, the mire coverage ranges from 0 to 44% across the sub-catchments, while the forest cover ranges from 54 to 99%. Those ecosystem types are well represented across the northern Swedish landscape (Table S1). The bedrock is dominated by metasediment/metagraywacke (94%). The catchment is weakly affected by agriculture (2%).

The stream sampling conducted in the ABI region spread across an area of more than 5 000 km^2^. The ABI region is located in the sub-arctic climate zone with the coldest climate of all four regions. The north-western part of the ABI region contains marble segments whereas the eastern part of the region is dominated by schist and quartzite bedrock. The vegetation is partially forested with the most alpine sections of the region falling above the tree line and covered with shrubs (Table S1).

**Table S1:** geographic and climatic properties of each studied region, presenting the mean annual temperatures (1961 to 1990 period), dominant lithology and landcover type, obtained from the Swedish Geographical Surve (SGU) and Lantmäteriet. Further details on the regional landscape properties are presented in ^10^ for LAVI and DAL, ^11^ for KRY and ^12^ for ABI.

| Region  Code | Sampling Design | Sampled  Streams | Area (km^2^) | Elevation  (mean ±SD)  (m.a.s.l.) | Mean Annual  Temperature  (C) | Mean Annual  Precipitation  (mm) | Mean Annual  Runoff  (mm) | Geology | Land Cover (%) |
| --- | --- | --- | --- | --- | --- | --- | --- | --- | --- |
| LAVI | Headwater Survey | 68 | 27 000 | 167 (±71)  0 to 373 | 4.5 to 6.5 | 750 to 1250 | 250 to 650 | Granite (87%), Rhyolite (3%),  Sandstone (2%), Other (8%) | Forested (61%)  Clear cuts (8%)  Peatlands (4%)  Lakes and ponds (7%)  Urban or Agricultural (20%) |
| DAL | Headwater Survey | 101 | 66 000 | 325(±235)  0 to 1321 | 0.5 to 4.5 | 650 to 950 | 250 to 550 | Granite (54%), Rhyolite (10%),  Sandstone, (14%), Other (22%) | Forested (67%)  Clear cuts (11%)  Peatlands (9%)  Lakes and ponds (6%)  Urban or Agricultural (7%) |
| KRY | Nested Catchment | 108 | 68 | 114 to 405 | 1.8 | 614 | 311 | Metasediment/metagraywack (94%)  Acid and intermediate metavolcanic rocks (4%)  Basic metavolcanic rocks (3%) | Forested (87%)  Clearcuts (7%)  Peatlands (9%)  Urban or Agricultural (2%)  Others (8%) |
| ABI | Nested Catchment | 49 | 5 800 | 708 (±301)  316 to 1959 | -1.21 | West :1000 mm  East: 400 mm | NA | Northwest: Mica schist with segments of marble  Southeast: schist and quartzite | Alpine (8%)  Transition vegetation (15%)  Forested (60%)  Peatlands (4%)  Lakes and Ponds (11%)  Permanent snow (1%)  Urban & Agricultural (1%) |

**Section 4: Supplementary results**

**Figure S2**: Kendall correlation matrix of stream water chemistry variables across the DAL, LAVI, KRY and ABI regions in Sweden.

**Figure S3**: Structural matrix of linear regression models between stream pH, C concentrations, δ ^13^C-DIC and the calculated δ ^13^C-CO_2_ across all streams The number’s associated to each arrow represent the adjusted R^2^ between two variables. All relationships were significant.

**Table S2**: Summary table of Dunn's test non-parametric pairwise multiple comparison z test statistic, asterisks indicate groups where a given variable is not significantly dissimilar between two regions (p-value >0.05).

|  |  | ABI | DAL | KRY |
| --- | --- | --- | --- | --- |
| δ^13^C-DIC  (‰) | DAL | -5.31 |  |  |
|  | KRY | -10.17 | -5.99 |  |
|  | LAVI | -13.29 | -9.95 | -4.58 |
| Modelled  δ^13^C-CO_2_  (‰) | DAL | -4.93 |  |  |
|  | KRY | -7.61 | -3.59 |  |
|  | LAVI | -11.84 | -9.27 | -6.06 |
| DOC  (mg C L^-1^) | DAL | 6.25 |  |  |
|  | KRY | 9.80 | 4.32 |  |
|  | LAVI | 14.19 | 10.02 | 6.28 |
| DIC  (mg C L^-1^) | DAL | -2.35 |  |  |
|  | KRY | -5.92 | -4.61 |  |
|  | LAVI | -5.44 | -4.03 | 0.03* |
| CO_2_  (mg L^-1^) | DAL | 6.71 |  |  |
|  | KRY | 8.05 | 1.58* |  |
|  | LAVI | 8.47 | 2.66 | 1.29* |
| pH | DAL | -6.18 |  |  |
|  | KRY | -11.36 | -6.36 |  |
|  | LAVI | -11.97 | -7.44 | -1.85 |
| Alkalinity (mmol L^-1^) | DAL | -4.05 |  |  |
|  | KRY | -9.16 | -6.13 |  |
|  | LAVI | -10.77 | -8.37 | -3.01 |
| Ca^2+^ (mmol L^-1^) | DAL | -6.58 |  |  |
|  | KRY | -9.04 | -3.01 |  |
|  | LAVI | -7.73 | -1.94 | 0.72* |
| Ca:Na | DAL | -5.63 |  |  |
|  | KRY | -6.88 | -1.50 |  |
|  | LAVI | -9.42 | -5.05 | -3.76* |

**Table S3:** List of the range of initial conditions used for the different model runs of δ^13^C-DIC changes by CO_2_ degassing from streams for individual regions, presenting the DIC concentration (mgC L^-1^), pH and δ^13^C-DIC values.

| Region | Parameter | DIC  (mg C L^-1^) | pH |
| --- | --- | --- | --- |
| LAVI | Min | 3.57 | 4.00 |
|  | Max | 6.08 | 5.00 |
|  | Avg | 4.66 | 4.81 |
| KRY | Min | 8.08 | 4.00 |
|  | Max | 45.64 | 5.06 |
|  | Avg | 28.95 | 4.41 |
| DAL | Min | 20.11 | 4.73 |
|  | Max | 45.64 | 5.69 |
|  | Avg | 38.25 | 5.24 |
| ABI | Min | 13.24 | 4.64 |
|  | Max | 40.12 | 6.67 |
|  | Avg | 26.42 | 5.73 |

**Figure S4:** Scatterplot showing the relationship between Ca^2+^ and HCO^-^_3_ Na normalised molar ratio in the dissolved phase. End-member reservoirs for carbonate, silicate, rainwater sources of bicarbonate ions and Ca^2+^ were taken from Gaillardet et al, 1999^13^. Grey circles represent the data from world’s largest rivers, also presented in Gaillardet et al, 1999^13^ while the other circles represent the data from this study, coloured according to their regions (DAL, LAVI, KRY and ABI).


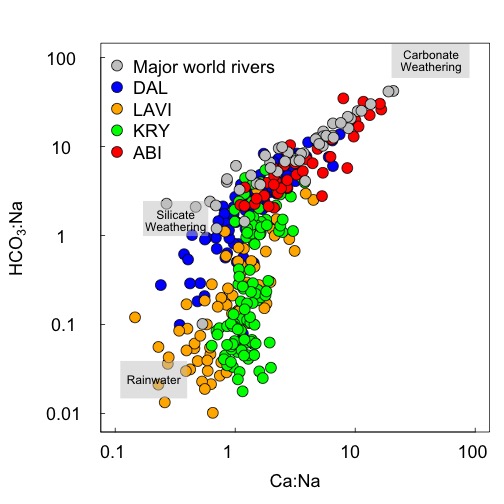


**Base Cation ratios across Swedish streams**. The comparison of base cation ratios (e.g. Ca:Na vs HCO_3_:Na) is a classic tool for distinguishing sources of carbonate alkalinity that was presented by Gaillardet, et al^. 13^ in a global study of world’s major rivers (Figure S3). It is interesting to note that the normalized base cation concentrations and alkalinity from Sweden covered the full range of reported values in this original study (Figure S3). Analysis of the Na normalised Ca^2+^ and HCO_3_^-^ molar ratio showed that the streams in ABI were closest to the carbonate weathering end-member, whereas the streams in DAL corresponded more closely to the silicate weathering end-member (Figure S3). The streams in LAVI and KRY, which contained very little alkalinity, overlay neither of those two end-members (Figure 4). The Ca concentrations were best correlated with alkalinity (r=0.60) and pH (r=0.59) (Figure S1). The Ca:Na ratio, which can be interpreted as an indicator of weathering sources, varied over two orders of magnitude (from 0.14 to 16.49) among all streams (Figure S3). The major world rivers dataset, presented in Gaillardet, et al^. 13^, was superimposed with our dataset to show the extent of the water chemistry gradient among the Swedish stream (Figure S3).

**Figure S5**: Boxplots summarizing the δ^13^C-DIC values (‰) for different stream orders in the ABI (red) and KRY (green) region where stream order >1 are also included. Boxes represent the median, and lower and upper quartiles (25% and 75%), while the whiskers show the 95% percentiles with minimum and maximum values shown as circles. Numbers of observations are specified below the whiskers for KRY and above for ABI.

**Figure S6**: Boxplots summarizing the δ^13^C-DIC values (‰) for different sampling occasion in the KRY region where sampling of the same subset of streams occurred at 7 different occasion. Boxes represent the median, and lower and upper quartiles (25% and 75%), while the whiskers show the 95% percentiles with minimum and maximum values shown as circles. Numbers of observations are specified above the whiskers.

**References**

1. Uppsala University, Department of Earth Sciences, Air Water and Landscape Sciences, Villavägen 16, SE-752 36, Uppsala, Sweden.

   *Correspondence: Audrey Campeau, Tel. 46+ 18-4712755, Fax. 46+ 18-55 11 24, email: audrey.campeau@geo.uu.se

   ^2^ Department of Geography and Environmental Studies, Wilfrid Laurier University, Waterloo, Ontario, Canada

   ^3^ Climate Impacts Research Centre, Department of Ecology and Environmental Science, Umeå University, Abisko, Sweden.

   ^4^ Department of Aquatic Sciences and Assessment, Swedish University of Agricultural Sciences, Uppsala, Sweden

   ^5^ Geology and Geochemistry, Stockholm University, Stockholm, Sweden.

   ^6^ Department of Earth and Environmental Sciences, University of Waterloo, Waterloo, Ontario, Canada [↑](#footnote-ref-2)
